# 2021 Acknowledgment of *mBio* Invited Editors

**DOI:** 10.1128/mbio.03681-21

**Published:** 2021-12-21

**Authors:** Arturo Casadevall

**Affiliations:** a Johns Hopkins Bloomberg School of Public Health, Baltimore, Maryland, USA

## EDITORIAL

On behalf of the editors of *mBio*, I gratefully acknowledge the following individuals, who served as invited editors for the journal in 2021. While not members of the Board of Editors, invited editors serve an important role in the review process. Invited editors are experts in their fields of research who add an additional level of quality to the review process. An editor may assign a paper to an invited editor when he/she would like to have an additional expert opinion of the reviews or when the subject area falls outside the editor’s primary area of expertise.

Mohamed Abdel-Mohsen
Michael W. W. Adams
Ramesh K. Akkina
Rebecca Alexander
Hal Alper
Galit Alter
Matthew Zack Anderson
David R. Andes
Nancie M. Archin
Eric Baehrecke
Nathalie Balaban
Megan Tierney Baldridge
Iliana Baums
Anke Becker
Gordon M. Bennett
Ira J. Blader
Joseph Bondy-Denomy
Marc J. Bonten
William J. Britt
Eric Brown
Tricia H. Burdo
Mary Carrington
Marina Caskey
Roberto Cattaneo
Arnab Chatterjee
Genhong Cheng
Shu-Sin Chng
Beatrice Clouet-d’Orval
John M. Coffin
Richard C. Condit
Brian Conlon
Francois-Loic Cosset
Anthony L. Cunningham
Cameron R. Currie
Zachary D. Dalebroux
Andrew J. Darwin
Kristen M. DeAngelis
William H. DePas
Ulrich Desselberger
Santosh Dhakal
Dirk P. Dittmer
Ray Dixon
Ivan Dorso
David Dubnau
Anne K. Dunn
W. Paul Duprex
Ashlee M. Earl
Lynn W. Enquist
Robert K. Ernst
Michael J. Federle
Mario F. Feldman
David A. Fidock
Erol Fikrig
Scott G. Filler
Sabine Fillinger
Alain Filloux
Peter Charles Fineran
Erik K. Flemington
Ana Flores-Mireles
Genevieve G. Fouda
Matthew B. Frieman
Isabelle Fudal
Deborah H. Fuller
Daniel J. Gage
George Fu Gao
Ferran Garcia-Pichel
Adam P. Geballe
Caroline Attardo Genco
Benjamin Gewurz
Douglas P. Gladue
Mark Goulian
Haitao Guo
Sara Hallin
Lynn E. Hancock
J. Marie Hardwick
Brian P. Hedlund
Emily Hemann
Jennifer K. Herman
John Hiscott
Chris Todd Hittinger
Erik Holmqvist
Johannes Huebner
William W. Ja
Ilse D. Jacobsen
Guilhem Janbon
Rohit K. Jangra
Kristina Jonas
Maria Kalamvoki
Robert F. Kalejta
Martin Kaltenpoth
Salim Abdool Karim
Andrew L. Kau
Yoshihiro Kawaoka
Mary F. Kearney
Thomas E. Kehl-Fie
Libusha Kelly
Melissa M. Kendall
Linda J. Kenney
Megan Kiedrowski
Kami Kim
Phillip E. Klebba
Robyn S. Klein
Sabra L. Klein
Julia Ruth Köhler
Jay Kolls
Erika Kothe
Laurie T. Krug
Laimonis A. Laimins
Chia Y. Lee
Petr G. Leiman
Petra Anne Levin
Henry Levin
Stuart M. Levitz
Sharon R. Lewin
Ruth E. Ley
Dominique H. Limoli
Steven E. Lindow
Jodi A. Lindsay
Gianni Liti
Shan-Lu Liu
Nathan Lo
Allison Lopatkin
Ricardo O. Louro
Anice C. Lowen
R. Craig MacLean
Nicholas J. Mantis
Maria L. Marco
Malcolm A. Martin
Michael J. McCullough
Aaron P. Mitchell
Masatoshi Miyakoshi
Tim Miyashiro
Luis J. Montaner
Walther Mothes
Karl Münger
Julia Oh
Michal A. Olszewski
Eng Eong Ooi
Tracy Palmer
Andrew Pekosz
Stanley Perlman
Yuri Persidsky
Mariagrazia Pizza
Vicente Planelles
Paul J. Planet
Damian Francis John Purcell
Sumant Puri
Lakshmi Rajagopal
Murugesan V. S. Rajaram
Matthew Mark Ramsey
Reeta Prusty Rao
John F. Rawls
Stuart C. Ray
Ranjit Ray
Michael L. Reese
Federico Rey
Andrew Rice
Erle S. Robertson
Kelly Ruggles
Carmen San Martín
Larry S. Schlesinger
Lutz Schmitt
Leon Schulte
Jessica Scoffield
David R. Sherman
Nicolas Sluis-Cremer
Victor Sourjik
Gerald Spaeth
Alfred M. Spormann
William J. Steinbach
Timothy P. Stinear
Paul D. Straight
Osamu Takeuchi
Yinjie J. Tang
Jeffery K. Taubenberger
Renee M. Tsolis
Cagla Tukel
Juan Esteban Ugalde
Jorge E. Vidal
Jose Luis Villarreal-Camacho
Daniel E. Voth
Jennifer Walker
Xin Wang
Digby F. Warner
Martin J. Warren
Christopher M. Waters
James Weger-Lucarelli
Matthew D. Weitzman
Ronald C. Wek
Haitao Wen
Duncan W. Wilson
Malcolm E. Winkler
Joshua J. Woodward
R. Mark Wooten
Jie Xiao
Peng Yuan
Kwok-Yung Yuen
Patricia Zambryski
Kateryna Zhalnina
Qiwei Zhang
Wenjun Zhang
Zhi-Ming Zheng
Hong Zhou

